# Exploring the relationship between intestinal flora and the pathological mechanism of myopia in adolescents from the perspective of Chinese and Western medicine: A review

**DOI:** 10.1097/MD.0000000000033393

**Published:** 2023-03-24

**Authors:** Xiaoming Xi, Liang Han, Mengmeng Ding, Jinglu Li, Chenye Qiao, Zongjian Liu, Shuyan Qie

**Affiliations:** a Rehabilitation Center, Beijing Rehabilitation Hospital, Capital Medical University, Shijingshan, Beijing, China; b School of Rehabilitation Medicine, Shandong University of Traditional Chinese Medicine, Jinan, Shandong Province, China; c School of Beijing Rehabilitation Medicine, Capital Medical University, Shijingshan, Beijing, China; d Beijing Rehabilitation Hospital, Capital Medical University, Shijingshan, Beijing, China.

**Keywords:** adolescent myopia, Chinese medicine, intestinal flora, pathological mechanism, prevention, treatment

## Abstract

The etiology of adolescent myopia involves genetic and environmental factors. The pathological mechanism of modern medicine includes blood perfusion, changes in blood molecules, neurotransmitters, and sclera remodeling. Chinese medicine believes that myopia is mainly related to the deficiency of liver blood and spleen and stomach disorders. The prevention and treatment of myopia in adolescents are very important, but in terms of the current incidence of myopia in adolescents and the level of clinical diagnosis and treatment, its prevention and treatment are insufficient. Modern medicine and traditional Chinese medicine both pay attention to integrity, so adolescent myopia should not only pay attention to eye changes but also pay attention to other body systems and other aspects of change. Intestinal flora has become a research hotspot in recent years, and it has been found that it is closely associated with multi-system and multi-type diseases. No studies have directly investigated the link between Intestinal flora and myopia in adolescents. Therefore, by summarizing the pathological mechanism of adolescent myopia and the connection between intestinal flora and the pathological mechanism of adolescent myopia, this paper analyzes the possible pathological mechanism of the influence of intestinal flora on adolescent myopia, providing a theoretical basis for future studies on the correlation between changes of intestinal flora and its metabolites and the incidence of adolescent myopia, which is of great significance for the study on the risk prediction of adolescent myopia.

## 1. Introduction

Myopia, a refractive abnormality, is a global public health problem that produces blurred vision due to an increase in the axial length of the eye,^[1]^ resulting in an elongated focal ratio that focuses light from distant objects in front of the retina.^[2]^ The prevalence of myopia is increasing globally, with a 20% prevalence in children under 6 years of age, with refractive abnormalities being the most common, followed by strabismus and amblyopia.^[1]^ Severe pathological myopia is closely associated with other ocular diseases and is usually determined by a combination of genetic and environmental factors.^[3]^ Juvenile myopia is the most common type of myopia, with the highest prevalence in grades 2, 5, and 6 of elementary school, and progresses rapidly during adolescence.^[2]^ The most typical risk factor for adolescent myopia is close work (e.g., reading, studying, television viewing, computer use, etc), but longitudinal studies have not confirmed that close work is the main cause of adolescent myopia.^[4]^ The treatment of myopia in adolescents is mainly optical correction and pharmacological intervention,^[5]^ but the results are not satisfactory, so prevention of myopia occurrence is the key. At present, there is no effective method to substantially prevent the occurrence of myopia, except for maintaining the correct study posture, limiting the time of electronic product use, eye exercises, and other healthy eye care science propaganda, which is related to the insufficient means of monitoring myopia risk factors. The intestinal flora is a hot spot for research and has been shown to regulate the systemic organs and tissues in both directions through the brain-intestinal axis, lung-intestinal axis, and kidney-intestinal axis, involving a wide range of diseases. The intestine is an important organ for nutrient absorption, and all tissues in the body need nutrient support to maintain normal physiological activities, so it is easy to understand that intestinal flora is closely related to multi-system and multi-species diseases. However, there are no studies that directly investigate the association between intestinal flora and myopia in adolescents.

## 2. Pathogenesis of myopia in adolescents

Although myopia is a pathological change of the eye, the pathological mechanism is not limited to the eye and surrounding tissues but may be associated with multiple systemic diseases in the body. This article summarizes the pathogenesis of myopia from both modern medicine and Chinese medicine.

### 2.1. Modern medical pathogenesis

The modern medical pathogenesis of myopia is complex, mainly including scleral remodeling, scleral hypoxia, related factor changes, dopamine mechanism, choroidal blood perfusion deficiency, and inflammatory response,^[6]^ etc, and each mechanism is interconnected and interacts with each other to influence the occurrence and development of myopia.

#### 2.1.1. Scleral remodeling.

Inappropriate extension of the eye axis is associated with scleral extracellular matrix remodeling that can cause a decrease in scleral strength and thickness, and is associated with slower synthesis and accelerated degradation of scleral extracellular matrix components,^[7]^ and a decrease in scleral extracellular matrix components weakens the scleral framework, resulting in a lengthening of the eye axis. For example, decreased expression of type I collagen, a major extra-scleral membrane component, weakens the structural framework of the sclera. Although a large number of experiments have supported this conclusion, the initiators and signaling pathway mechanisms that trigger these changes are not uniformly established but are thought to be mainly related to the hypoxia signaling pathway, eIF2 signaling pathway, and mTOR signaling pathway. Among them, the eIF2 signaling pathway and mTOR signaling pathway are in turn related to scleral hypoxia,^[8]^ and the 3 interact with each other. In addition, the choroid can translate and transmit myopic visual signals to the sclera, which may affect scleral extracellular matrix remodeling and ocular growth.^[7]^ However, the mechanisms regulating ocular development between the choroid and sclera are unclear.

#### 2.1.2. Scleral hypoxia.

Since hypoxia is a risk factor for pathological myopia, 1 study^[8]^ used the Fluidigm C1 System, and determined the transcriptomes of 93 single cells isolated from form-deprivation (FD) scleras; untreated fellow eyes served as control. Forty-nine of the cells were from 4 scleral samples (each sample contained 6 to 8 scleral tissues) of FD eyes; 44 cells originated from 3 samples (from similarly pooled scleras) of control eyes. Determined the dynamics of hypoxia-inducible factor 1α (HIF-1α) protein levels in mice, an indicator of tissue hypoxia, after 2 days and 2 weeks of FD, and compared to controls, form-deprivation myopia (FDM) had significantly higher scleral HIF-1α levels compared to controls, and increased axial elongation was observed after 2 weeks. This suggests that local scleral hypoxia occurs during the development of myopia. To further investigate the role of scleral hypoxia in myopia, this study used immunoblotting to discover that myofibroblasts in human scleral fibroblasts (HSFs) underwent trans differentiation and collagen production after reducing ambient oxygen levels to 5%; in HSFs exposed to hypoxic conditions, HIF-1α protein, adherent spot protein, and emyofibre increased expression of the fibroblast marker α-smooth muscle actin and decreased expression of type I collagen α1 protein in HSFs exposed to hypoxic conditions.^[8]^ The previous section discussed that collagen production weakens the scleral structural framework, so scleral hypoxia may lead to myopia through scleral myofibroblast trans differentiation and cause scleral remodeling.

#### 2.1.3. Choroidal blood perfusion.

The metabolic and nutritional processes of the body’s tissues are dependent on blood perfusion,^[9]^ and if blood perfusion is insufficient, it can lead to tissue ischemia, hypoxia, and even necrosis, affecting the development of the body. Studies have shown that visual signals lead to a decrease in choroidal capillary patency and blood flow, which leads to a decrease in the level of oxygen and nutrient supply to the adjacent sclera with no vascular distribution and consequent scleral hypoxia, which promotes myofibroblast trans differentiation through the accumulation of HIF-1αwhile reducing collagen production.^[10]^ Since the sclera has no vascular distribution, insufficient choroidal blood perfusion can indirectly lead to scleral hypoxia, which triggers a series of pathological changes leading to the development of myopia. Based on the above findings, it can be hypothesized that in addition to visual signals that can lead to inadequate choroidal perfusion, the body’s blood deficiency (e.g., anemia) may also lead to reduced choroidal blood flow and thus myopia. To verify this hypothesis, we can collect children or adolescents who meet the diagnostic criteria of anemia as the observation group and normal children or adolescents as the control group, and observe the incidence of myopia in the 2 groups or follow up after a few years to see if there is a difference in the incidence, which can provide a background for later studies.

#### 2.1.4. Dopamine mechanisms.

Dopamine (DA) is an important neurotransmitter in the retina that mediates a variety of functions including development, visual signaling, and refractive development.^[11]^ Since the first discovery of a link between DA and ocular growth control in 1989, more and more studies have adopted the DA hypothesis (i.e., that the retina releases DA to counteract myopic development) as the main hypothesis for myopia control. Light control methods such as outdoor activities may be used to suppress myopia through DA-mediated mechanisms,^[12]^ such as controlling retinal DA signaling and promoting refractive development.^[13]^ Enhanced neuronal activity maintains homeostatic storage of DA,^[11]^ so neuronal activity with DA as a hub is associated with normal retinal development, and reduced neuronal activity may inhibit retinal refractive development, etc, and myopia occurs. Neurons and DA are the basic units and important molecules of the nervous system, respectively, so the relationship between myopia and the nervous system can be studied in depth. Studies have shown that increasing DA content by injecting DA directly into the eye or using levodopa to promote DA synthesis, or enhancing DA signaling by nonselective DA receptor agonists such as APO and 2-amino-6, 7-dihydroxy-1, 2, 3, 4-tetrahydronaphthalene hydrobromide can prevent the development of myopia.^[11]^ Therefore, reduced DA content is an important cause of myopia, which may specifically include reduced DA synthesis, reduced release, and increased destruction, or DA receptors affected resulting in reduced DA content. DA may inhibit myopia by triggering the release of other transmitters from the retina or choroid and inducing choroidal thickening.^[14]^DA receptors are present in almost all neuron types in the retina, including D1, D2, D4, and D5 receptors, and are also associated with other intracellular signaling pathways. D1 and D5 receptors (D1-like receptors) promote intracellular cyclic adenosine monophosphate (cAMP) synthesis, while D2 and D4 receptors (D2-like receptors) inhibit cAMP synthesis.^[15]^ cAMP promotes DA synthesis, so D1 and D5 receptors can increase DA levels to inhibit myopia, and their deficiency may be a risk factor for myopia; D2 and D4 receptors have opposite roles to D2 and D5 in maintaining DA levels and increased D2 and D4 can reduce DA levels to induce myopia, which may be a risk factor for myopia. It was demonstrated that D2-like receptor antagonists inhibited the transient protective effect on unrestricted vision in form-deprived myopia, but not in negative crystal-induced myopia.^[16]^ This result suggests that DA may prevent the development of myopia by inhibiting form-deprivation and lens defocus.

#### 2.1.5. Associated factors.

Related factors act as intermediate bridges involved in the development and progression of myopia. They include trans differentiation of HIF-1α and HSFs, increased expression of matrix metalloproteinase-2 and α-smooth muscle actin, and decreased expression of type I collagen α1 protein, which jointly affect myopia through a bidirectional effect with scleral hypoxia and scleral remodeling. Among them, HIF-1α has been studied more frequently and is also the main relevant factor affecting myopia. In 1 study, a total of 30 patients (13 males, 17 females, age: 24–50 years) without pathologic myopia and underwent physical examination at The University of Hong Kong-Shenzhen Hospital was selected for collection of fasting peripheral blood as a normal control. Besides, the fasting peripheral blood of 30 patients (15 males, 15 females, age: 22–50 years) diagnosed with pathologic myopia in the ophthalmology department of The University of Hong Kong-Shenzhen Hospital was collected as the experimental group. Used microarray-based pathological myopia gene expression profiles to identify differentially expressed genes and cultured HSFs under hypoxic conditions, and showed that HIF-1α reduced miR-150-5p expression and promoted LAMA4-mediated activation of the p38 MAPK signaling pathway, which prevented extracellular matrix degradation in HSFs, ultimately leading to myopia.^[17]^ In another experiment, guinea pigs were randomly divided into 2 groups (30 guinea pigs in each group): FDM group and control group (without any treatment). Used a monocular form-deprivation method to induce high myopia in guinea pigs, and a 532 nm laser was used to induce choroidal neovascularization (CNV) generation; the results showed that CNV proliferation in the FDM group was proportional to time, the incidence of proliferation was higher than that in the control group, and the hypoxia-inducible factor HIF- 1α and vascular endothelial growth factor (VEGF) expression were increased in both groups, and in addition, miRNA-21 expression was positively correlated with VEGF and HIF-1α expression in both groups.^[18]^ Therefore, it can be concluded that CNV causes severe damage to central vision by a mechanism in which miRNA-21 is associated with the HIF-1α-VEGF signaling pathway, possibly by promoting the formation of CNV in guinea pigs and thus leading to myopia development.

#### 2.1.6. Inflammatory response.

Dysregulation of inflammatory pathways may contribute to the pathogenesis of myopic retinal degeneration, specifically including upregulation of the AGE-RAGE signaling pathway, complement cascade, NOD-like receptor signaling pathway, IL-17 signaling pathway, and TNF signaling pathway, while downregulation of antigen processing and cell adhesion pathways.^[19]^ In addition, CCL2,^[20]^ IL-6, matrix metalloproteinase-2, and angiopoietin-1 levels are higher in highly myopic eyes.^[21,22]^ It can be seen that the inflammatory response may be involved in the development and progression of myopia through altered signaling pathways and the production of inflammatory factors, but it is unclear whether the inflammatory response is a consequence or a cause of myopic retinal degeneration.

#### 2.1.7. Other.

Myopia is also associated with ocular degenerative changes, including posterior chylomicrons, lacunar fissures, optic disc abnormalities, and choroidal atrophy,^[23]^ which may result from ocular neuronal dysfunction or apoptosis. In addition, women have risk factors for high myopia^[24]^ and there may be gender differences in the development of myopia,^[25]^ so it is speculated that there may be some degree of relationship between estrogen and myopia.

### 2.2. Pathogenesis in Chinese medicine

Myopia is referred to in Chinese medicine as “being able to be near and timid to be far,” and its symptoms were first recorded in the Treatise on the Origin of Diseases, and also described in the “Zhengzhi Zhunsheng - The 7 Focal Points” and “Dacheng - Volume 2 of the Eye Classic.” However, the relevant TCM pathogenesis has been recorded as early as in the Yellow Emperor’s Classic of Internal Medicine. According to TCM, myopia is mainly related to liver and blood deficiency, congenital endowment deficiency, acquired spleen, and stomach dysregulation, and Yang deficiency.^[26]^ According to Su Wen - Generation of the 5 Organs, “All the veins belong to the eyes...... blood goes to the liver, and the liver receives blood and can see.” It is pointed out that the function of the eyes depends on liver blood to moisten them, and the liver opens the orifice of the eyes, and the eyes are closely related to the liver. Therefore, a deficiency of liver blood can cause the eyes to lose blood to moisten them, resulting in symptoms such as dry eyes and blurred vision. The Ling Shu points out that “the vein of the liver and the foot of the Fructus yin...... goes up into the forehead and connects to the eye system......,” which believes that the liver has a close connection with the eyes through the liver meridian. The Yellow Emperor’s Classic of Internal Medicine states that “the liver and kidney are of the same origin,” or “the essence and blood are of the same origin,” and that the kidney is the master of bone, bone produces marrow, and marrow produces blood. The spleen and the stomach are the origins of the latter, the source of biochemical energy and blood, and the blood of the whole body is based on the biochemical blood function of the spleen and stomach. If the spleen and stomach are dysfunctional, blood biochemistry will be blocked, which will not only lead to dysfunction of the internal organs and meridians but also dysfunction due to the loss of blood moistening. The Sibai point in the stomach meridian is located 1 inch directly below the pupil of the eye and has the function of treating blindness in the eyes. The Ling Shu states: “The vein of the small intestine of the hand sun...... follows the neck up the cheek from the absence of the basin to the eye sharp canthus...... not the cheek on the against the nose, to the eye inner canthus......,” it can be seen that the small intestine has a very close connection with the eyes through the small intestine meridian. The Yellow Emperor’s Needle Classic suggests that both the large intestine of the hand Yangming and the small intestine of the hand Sun belong to the stomach of the foot Yangming, and from the analysis of the meridian connection and the physiological functions of the large and small intestines and the stomach, it is also suggested that the large and small intestines are attached to the stomach.^[27]^ Therefore, dysfunction of the spleen and stomach is one of the important pathogenic mechanisms of myopia. Studies have shown that outdoor activities can inhibit the occurrence of myopia, and people with Yang deficiency tend to be mentally uninspired, quiet, and introverted, and mostly dislike outdoor sports,^[28,29]^ so the incidence of myopia may be high. In addition, the function of blood to moisten the organs and tissues is diminished in Yang deficiency patients, so Yang deficiency patients have the conditions for myopia to occur.

## 3. Linkage between intestinal flora and the pathogenesis of myopia in adolescents

### 3.1. Modern medical connections

Through literature reading, it is found that intestinal flora is related to tissue ischemia and hypoxia, dopamine alteration, and inflammatory response, and these mechanism alterations are exactly the pathological mechanism of myopia, but there are no studies directly explore the connection between intestinal flora and myopia pathological mechanism, so this paper will analyze from this aspect.

#### 3.1..1. Intestinal flora and tissue ischemia and hypoxia.

Recent studies have shown that oxygen dynamics plays an important role in regulating homeostasis in the intestine and has a bidirectional regulatory mechanism with the intestinal flora.^[30]^ The intestine is highly dependent on adaptive pathways activated by hypoxia, and HIF-1α is an important hypoxic factor, so HIF-1α is inextricably linked to the intestinal flora. Then, intestinal flora may weaken the scleral structure by increasing HIF-1α levels, leading to myopia development. Studies have shown that disorders of intestinal flora can contribute to the development of ischemic stroke or aggravate cerebral ischemia by enhancing systemic inflammation.^[31]^ A non-randomly control study by Fu Ke et al^[32]^ intervened in the intestinal flora of patients with cerebral infarction by Astragalus Stomach Zhenzhu Pill and found that it reduced cerebral ischemia-reperfusion injury in patients with cerebral infarction. This further suggests that intestinal flora disorders can contribute to the development of cerebral ischemia. The choroidal artery (chA) is divided into the anterior choroidal artery (AchA), the medial posterior choroidal artery, and the lateral posterior choroidal artery. AchA originates from the internal carotid artery (The AchA originates from the internal carotid artery, the medial posterior choroidal artery from the P1 or P2a segment of the posterior cerebral artery, and the lateral posterior choroidal artery from the P1 or P2a or P2p segment of the posterior cerebral artery. It can be seen that the chA is closely connected to the blood supply arteries of the brain, and poor circulation in the brain can lead to insufficient blood in the chA. Insufficient blood in the chA can have a direct effect on vision. The intestinal flora is closely related to the blood supply of the brain, so there is an indirect connection between the intestinal flora and the blood supply of the chA, so it is theoretically very likely that there is a close connection between the intestinal flora and the visual acuity. This inference can guide the basic experimental research on the relationship between intestinal flora and ocular hemodynamic changes and visual acuity, which, if confirmed experimentally, will be of great significance for both theoretical research on the pathogenesis of myopia and clinical treatment research.

#### 3.1.2. Intestinal flora and dopamine alterations.

The relationship between gut flora and dopamine has become a hot topic of research in recent years. It has not been fully demonstrated that intestinal flora regulates dopamine in vivo, but there is growing evidence that it may play a role in host biosynthesis or catabolism.^[33]^ The existence of a phenylalanine-tyrosine-dopa-dopamine metabolic pathway in microorganisms^[34]^ suggests that bacteria may contain homologs of the enzyme genes used by mammals to produce dopamine,^[35,36]^ such as enterococci for example, which produce biogenic dopamine.^[37]^ Tyrosine hydroxylase is a key enzyme in the dopamine synthesis pathway.^[38]^ It was shown that oral administration of flavopiridol triggered the biosynthesis of BH4 in the intestinal flora, increased the concentration of dopa/dopamine in the blood and brain, enhanced tyrosine hydroxylase activity to produce L-dopa, and ultimately improved animal fitness; in contrast, oral antibiotics reduced the number of intestinal flora and thus the ability of the intestine to produce dopamine.^[34]^ This further suggests that intestinal flora can promote dopamine synthesis and release into the bloodstream, where dopamine runs to the brain and other parts of the body to function. Dopamine is an important neurotransmitter in the retina, which is closely related to vision, and its deficiency can lead to abnormal visual development and vision loss.^[11]^ Therefore, intestinal flora dysbiosis could theoretically lead to visual development and vision loss by inhibiting dopamine synthesis and decreasing dopamine concentration in the retina. There are no studies to investigate the relationship between intestinal flora and visual acuity, and this paper uses dopamine as a bridge to establish the link between intestinal flora and visual acuity, which can improve a new direction for basic research on the pathogenesis of visual loss. It is recommended that related basic research observe which intestinal flora and how they change can lead to myopia through bioassay technology. This will not only be an important tool to detect the risk of myopia in adolescents but also may provide a new method to treat myopia in adolescents, which is important for the detection, prevention, and treatment of myopia in adolescents.

#### 3.1.3. Intestinal flora and inflammatory response.

Studies have shown that fermented foods increase intestinal flora diversity and decrease inflammatory markers.^[39]^ Gut flora translocation promotes the inflammatory response of the immune system and can lead to inflammation in some tissues, resulting in loss of function.^[40]^ This suggests that intestinal flora is closely related to the inflammatory response. Among them, CCL2, IL-6, IL-17, AGE-RAGE signaling pathway, and TNF signaling pathway are closely related to the intestinal flora,^[41]^ and these inflammatory factors and signaling pathways are precisely the pathological factors and signaling pathways of myopia, so changes in the intestinal flora may produce inflammatory responses through these inflammatory factors and signaling pathways and affect the development and progression of myopia. In addition, based on the intestine-hepatic axis theory, after the intestinal barrier is damaged, bacterial translocation endotoxin enters the portal system and activates liver blast cells, etc, and then releases a series of inflammatory factors that further aggravate liver damage and disease progression.^[42]^ The link between liver and vision loss was briefly summarized by Yu Zeng Fang et al^[43]^ For example, hepatic-brain-eye syndrome, a form of cortical blindness secondary to severe liver disease, often manifests as transient visual impairment; in functional magnetic resonance imaging of patients with mild hepatic encephalopathy, a reduction in blood oxygen level-dependent signals associated with the right parietal cortex of the brain and visual judgment was found, with the potential for cortical blindness; in lenticular In the retinal pigment epithelium of lens-induced myopia guinea pigs, stable expression and elevated activity of hepatocyte growth factor, a major cytokine derived from hepatocytes and stimulating hepatocyte proliferation, were found. This suggests that the liver and eye are closely related, and liver disease can lead to abnormal visual function, while hepatitis is the main cause of many liver diseases. Therefore, intestinal flora may affect vision through the intestine-liver axis and liver-eye pathological signaling pathways. In TCM, the liver opens the eyes and the liver-eye connection is very close, which provides the theoretical basis and academic background for the above inference.

### 3.2. Chinese medicine connection

The function of intestinal flora is similar to that of the spleen and stomach in Chinese medicine, such as the main digestion, absorption, and nutrient delivery, and has a close connection with the functions of the organs, tissues, organs, and meridians, which can be understood as the qi of the spleen and stomach and the yin and yang of the spleen and stomach.^[27]^ Therefore, the connection between intestinal flora and myopia can be converted into a connection between the spleen and stomach and myopia. The liver’s main blood collection function depends on the spleen and stomach’s biochemical function of qi and blood, and if the spleen and stomach function is impaired then the biochemical deficiency of qi and blood can directly lead to liver blood deficiency. The spleen and stomach are the basis of the postnatal essence, which can nourish the innate kidney essence. A deficiency of Yang in the spleen and stomach will not only lead to slow movement of Qi and blood, weakening the role of warming and nourishing local tissues but will also affect the normal physiological functions of other internal organs throughout the body. Therefore, abnormal function of the spleen and stomach can lead to pathological mechanisms closely related to myopia, such as liver and blood deficiency, innate endowment without nourishment, and yang deficiency, which can lead to myopia.

## 4. Summary and outlook

There is no study to investigate the relationship between intestinal flora and myopia. In this paper, by summarizing the pathogenesis of myopia, we found that there is an overlap with the pathological condition caused by intestinal flora, mainly in 3 aspects: tissue ischemia and hypoxia, dopamine alteration, and inflammatory response, so we think that the change of intestinal flora may lead to the occurrence of myopia. The relationship between the 2 was also analyzed from the perspective of Chinese medicine, providing an academic background and theoretical basis for this inference. It can provide a reference direction for research related to myopia in adolescents. If future research detects the relationship between intestinal flora changes and visual acuity, it will be of great significance for the risk detection, prevention, and treatment of myopia in adolescents.

## Author contributions

**Resources:** Xiaoming Xi, Mengmeng Ding, Jinglu Li, Chenye Qiao, Zongjian Liu, Shuyan Qie.

**Supervision:** Xiaoming Xi.

**Writing – original draft:** Xiaoming Xi.

**Writing – review & editing:** Xiaoming Xi, Liang Han.
